# Examining Associations between Body Mass Index in 18–25 Year-Olds and Energy Intake from Alcohol: Findings from the Health Survey for England and the Scottish Health Survey

**DOI:** 10.3390/nu10101477

**Published:** 2018-10-10

**Authors:** Viviana Albani, Jennifer Bradley, Wendy L. Wrieden, Stephanie Scott, Cassey Muir, Christine Power, Niamh Fitzgerald, Martine Stead, Eileen Kaner, Ashley J. Adamson

**Affiliations:** 1 Human Nutrition Research Centre, Newcastle University, Newcastle upon Tyne NE2 4HH, UK; viviana.albani@newcastle.ac.uk (V.A.); jen.bradley@newcastle.ac.uk (J.B.); cassey.muir@newcastle.ac.uk (C.M.); ashley.adamson@newcastle.ac.uk (A.J.A.); 2 Institute of Health and Society, Newcastle University, Newcastle upon Tyne NE2 4HH, UK; eileen.kaner@newcastle.ac.uk; 3 Fuse—the Centre for Translational Research in Public Health, Newcastle upon Tyne NE2 4HH, UK; S.J.Scott@tees.ac.uk; 4 School of Social Sciences, Humanities and Law, Teesside University. Middlesbrough TS1 3BA, UK; 5 Population, Policy and Practice, UCL Great Ormond Street Institute of Child Health, 30 Guilford Street, London WC1N 1EH, UK; christine.power@ucl.ac.uk; 6 Institute for Social Marketing, UK Centre for Tobacco and Alcohol Studies, Faculty of Health Sciences and Sport, University of Stirling, Stirling FK9 4LA, UK; niamh.fitzgerald@stir.ac.uk (N.F.); martine.stead@stir.ac.uk (M.S.)

**Keywords:** alcoholic beverages, obesity, young adults, recommended dietary allowances, body mass index, England, Scotland, health survey

## Abstract

Evidence on the relationship between alcohol consumption and body mass index (BMI) is mixed, particularly for young adults. This study explored the relationship between energy obtained from alcoholic beverages and BMI using data for 18–25 year-olds (*n* = 7691) from pooled cross-sections of the 2008–2014 Health Survey for England and the Scottish Health Survey. Energy obtained from alcoholic beverages (excluding mixers) on the heaviest drinking day in the past week was expressed as percentage of total recommended dietary allowance (RDA) of energy (% RDA Energy). Linear regressions were estimated of BMI on alcohol intake categories controlling for intake frequency, physical activity, longstanding illness and other covariates, with separate analyses for men and women, and by beverage type. Significant associations with BMI were observed with the ‘Very High’ category of alcohol intake (>75% RDA Energy) for men (*p* < 0.001, 1.74, 95% confidence interval (CI) 0.98, 2.49) and with the “High” (>50% to 75% RDA Energy) (*p* < 0.001, 1.67, 95% CI 0.26, 2.58) and above category for women, when compared with the Low (>0–25% RDA Energy) category. Young adults drinking the highest levels of alcohol on a single occasion were more likely to be obese than those with the lowest intake. Interventions to address internationally rising youth obesity rates should also consider reducing alcohol consumption by increasing alcohol prices, and reducing availability and marketing exposure.

## 1. Introduction

Increasing rates of overweight and obesity across adult populations globally pose an important challenge for public health authorities [1]. A high body mass index (BMI) is associated with increased risk of cardiovascular disease, type 2 diabetes, several types of cancer, and many other disorders which add to the global burden of chronic disabilities [2,3]. Moreover, these conditions or their precursors are more likely to affect young people who are overweight or obese [4]. Currently in England around 60% of all adults and one in three young people between the age of 16 and 24 years are obese or overweight [5], with more recent cohorts of young adults having a steeper increase in BMI than their predecessors [6,7].

The observed trends in excess body weight in young adulthood may be explained by the prevalence of risk behaviours for overweight and obesity between the ages of 18 and 25 years [8]. Among other factors, high alcohol intake may play an important role in weight gain during this period [9]. The potential relationship between alcohol intake and body weight arises from both biological and behavioural pathways. Biologically, alcohol contributes to increased fat stores in the body. Its metabolism inhibits lipid oxidation as one of its main metabolites, acetate, represents a readily available energy source that is used by the body in preference to fat; a phenomenon which is more pronounced with diets that are already high in fat and energy [10]. It has also been suggested that unhealthy food choices are more likely to be made during and directly after a period of alcohol consumption [11] which could, at least in part, be due to the disinhibiting effect of alcohol as a psychoactive substance that can alter usual behaviour [12]; or also because of the appetite enhancing effect of alcohol [13].

Behaviourally, alcohol consumption is typically accompanied by diets higher in fat and other risk factors for elevated adiposity, such as smoking and sedentary activities [14].

Evidence from epidemiological studies of the relationship between alcohol intake and body weight in adults is mixed. Whereas most studies have found an inverse relationship between overweight/obesity and more frequent drinking, evidence regarding the relationship with the amount of alcohol consumed is less clear [15,16,17,18,19]. At the same time, non-drinkers have been found to have higher BMI or obesity risk compared to drinkers, suggesting a non-linear relationship between alcohol and obesity [20,21,22]. So far, however, few studies have looked at the relationship between alcohol consumption and obesity in young adults, a point in the life course at which the relationship between BMI and alcohol intake may be less affected by reverse causation. For example, in older adults it may be difficult to know if alcohol consumption increases likelihood of being obese or being obese leads to high alcohol intake.

The type of alcoholic drink may show different relationships with BMI. Studies looking at adult populations (>18 years) have generally found no association between wine consumption and BMI and positive associations for beer and spirits, particularly in men [23], possibly reflecting broader lifestyle patterns for which spirits or beer are the preferred alcoholic beverages [19,24]. In the UK, wine is less popular than “white” spirits (e.g., vodka, gin, white rum and tequila) with 18 to 24 year-olds, and these spirits are more popular in this age group than with any other age group of consumers [24]. This preference may influence the shape of the BMI-alcohol intake association in young adults.

The relationship of alcohol consumption to body weight varies between men and women because of different cultural norms around alcohol [19,24] and because of differences by sex in alcohol metabolism that reflect differences in body fat between men and women [9]. In the following study, young adults were defined as individuals between 18 to 25 years of age. This age group has the heaviest concentration of acute alcohol harms such as accidents and assaults, in part because of social and environmental factors encouraging patterns of excessive drinking during this period [25,26]. Notably, these patterns of excessive drinking have the potential to set individuals onto drinking and overweight trajectories that persist into the rest of adult life [26,27,28]. Understanding the relationship between alcohol intake and BMI in young adults is therefore important to inform the wider debate on the effects of alcohol on young individuals’ health and well-being.

The aim of this study was to determine the relationship between alcohol intake, expressed as percentage of the gender-specific recommended dietary allowance (RDA) of energy (% RDA Energy) from alcoholic beverages (excluding mixers) on the heaviest drinking day in the last seven days, and BMI in young adults. Expressing alcoholic beverage intake as a proportion of energy and standardising as a proportion of gender specific recommendations for energy illustrates the consumption that could contribute to obesity.

## 2. Materials and Methods

### 2.1. Data

The study used two UK nationally representative population survey datasets, the Health Survey for England (HSE) and the Scottish Health Survey (SHeS), for the years 2008–2014. Full details of the methodology of these surveys are published elsewhere [29,30]. The HSE sampled around 8000 adults (16+) in each survey wave, with some variation by years, while the SHeS sampled around 6000 adults (16+) between 2008 and 2011, and 4000 adults in each survey year between 2012 and 2014 [29,30,31].

Content of the surveys included information on BMI (kg/m^2^), alcohol consumption and well-being. Data for analyses came from the combined 2008 to 2014 cross-sections of the HSE and SHeS. The actual survey years included in the study, however, varied depending on whether the variables of interest were collected on the different survey waves, seen in the online supplementary material in Table S1. The sample was restricted to young adults (*n* = 8805) aged between 18 and 25 years, inclusive. We looked at differences by types of alcoholic beverages, and in addition, at differences between men (*n* = 3838) and women (*n* = 4967).

### 2.2. Measures

BMI was estimated as weight (kilograms) divided by height (metres) squared measured by trained interviewers using the HSE and SHeS protocols [29,30,31].

For both surveys trained interviewers asked participants about their drinking habits and young adults aged 18 to 24 had in addition the option to use self-completion booklets to allow confidentiality of responses in front of other members of the household. Drinking frequency was recorded by asking about how frequently alcohol was consumed in the last 12 months with response categories from the original item in the survey grouped into “5 or more times a week”, “1 to 4 times a week”, “1 to 2 times a month”, “less than once every couple of months”, “used to drink and stopped”, and “never a drinker”. To assess the amount of alcohol consumed, participants were asked to report the number of days in the past 7 days in which they had an alcoholic drink and the specific type and amount consumed on the heaviest drinking day of that week.

Questions asked were about consumption of glasses of wine and sherry, pints or cans of beer (regular and strong strength), measures of spirits and bottles alcoholic soft drink (“alcopop”), or a pre-mixed alcoholic drink (Alcopops and pre-mixed alcoholic drinks are termed ready-to-drink (RTDs)). In addition, an indicator variable was derived to note if the individual drank more than one type of alcoholic drink on the heaviest drinking day, e.g., when reporting consumption of beer, if wine or spirits were also consumed, which acted as control for the potential confounding effect of different types of beverages consumed when assessing the relationship of BMI with the consumption of specific types of alcohol.

In this study the energy obtained from alcoholic drinks on the heaviest drinking day was calculated by multiplying the volume of the drink by the average kcal content (per mL) of the specific drink type times the number of drinks [32]. The kilocalorie (kcal) was used in preference MJ in this study due to the nature of the data available. The average energy content of broad categories needed for the study was only available as kcal and is shown in Table 1. This shows the contribution a single standard measure of popular alcoholic beverages would make to % RDA Energy for males and females, excluding any additional contribution from non-alcoholic drinks typically drunk in combination with spirits (“mixers”). A similar approach has been used by others [33] but not specifically for this age group. Taking the data on alcohol consumption from the HSE and the SHeS, the energy content included kcals from the whole drink (e.g., from carbohydrates), not just energy from alcohol, as seen in Table 1, and was estimated for each drink category, and for total alcohol consumption as the sum across categories. It was not possible to add in the contribution of any non-alcoholic mixer drinks such as tonic water or lemonade added to spirits; therefore, energy (kcals) from alcoholic drinks used in this analysis includes all energy from, for example, beer, wine, or gin rather than only energy from the alcohol within these drinks, but excludes energy from any mixers added to drinks.

The % RDA Energy (percentage of recommended daily energy allowance from alcoholic beverages consumed by individuals on their heaviest drinking day) was calculated for each individual by dividing their estimated alcohol kcal content by the relevant recommended daily energy allowance (RDA-2500 kcal for men and 2000 kcal for women [34]). For the analysis individuals were then grouped into categories according to their % RDA Energy as follows: None (0%); Low (>0–25%); Medium (>25–50%); High (>50–75%); and Very High (>75%) for total alcoholic drinks and beer. For wine, and spirits and RTDs the High and Very High Categories were combined (>50%).

The dataset included the age, sex and ethnicity of participants. The relationship between % RDA Energy and BMI may be confounded by other variables that are associated with alcohol intake and weight. Therefore, potential confounders included in analyses were employment status (level of education and socioeconomic status were also collected but employment status was used, as the relationship with BMI was the stronger of the three intercorrelated variables), being a parent, reporting a limiting longstanding illness, eating five or more portions of fruit and vegetables per day, smoking cessation, and level of physical activity. Physical activity was measured using a questionnaire [29,30,31]. Level of physical activity was categorised for consistency across surveys according to national recommendation thresholds as Low, Medium or High levels of moderate or vigorous physical activity (MVPA).

Recommendations for MVPA changed between 2008 and 2013 so it was necessary to recode earlier variables into MVPA tertile equivalents. For the 2008 HSE this variable was derived by grouping the categories of “Inactive” and “Lower but active” as Low, “30 min moderate physical activity five times per week” as Medium, and at least “30 min of vigorous physical activity three times a week” as High. Values in the 2012 HSE were recoded from “Inactive” and “Low activity” as Low MVPA, “Some activity” as Medium, and “Meets guidelines” as High. Physical activity data were not collected in the 2009, 2010, and 2011 HSE survey waves [35]. Smoking cessation is associated with weight gain [36], and to allow for this, an additional variable was analysed: whether individuals had quit smoking in the last 12 months. Other variables included in the analyses were survey year and country.

### 2.3. Statistical Analyses

Sample characteristics were summarized using measures of central tendency and dispersion for continuous variables, and frequency distributions for categorical variables. The relationship between alcohol consumption (in categories of % RDA Energy) and BMI was explored using linear regression controlling for the set of health, social and demographic characteristics. Confounders were selected based on a literature review based on previous studies looking at the relationship of alcohol and BMI as well as demographic factors influencing BMI level. The category of Low (>0–25% RDA) was used as a reference (25% of RDA is equivalent to just over half a litre (two large glasses) of wine for a women or one and a half pints (approximately 850 mL) strong beer for a man) (Table 1).

Regressions parameters were estimated separately for males and females, for % RDA Energy from total alcoholic beverage intake and by type of alcoholic beverage. In addition to the list of potential confounders, regressions for total alcoholic beverage intake controlled for frequency of intake in the last 7 days and over the last 12 months (including being a non-drinker), and regressions by alcohol type additionally incorporated the indicator variable for consuming more than one type of drink on the heaviest drinking day. Regression standard errors and confidence intervals were calculated using 1000 bootstrap replications stratifying by survey year. A *p*-value lower than 0.05 was considered evidence of a statistically significant relationship. All analyses were conducted using Stata statistical software version 15 (StataCorp LLC, College Station, TX, USA) [37].

## 3. Results

### 3.1. Sample Characteristics

Table 2 presents respondents’ characteristics in relation to alcoholic beverage consumption for None, Low, Medium, High and Very High % RDA Energy from alcoholic beverages on the heaviest drinking day. Almost 90% of respondents claimed to drink alcohol regularly or occasionally but not all were able to report alcohol intakes for the heaviest drinking day. There were more women (63%) than men (37%) in the None (0% of energy from alcohol) category, but more men (61%) than women (39%) engaging in very high alcohol consumption (>75% RDA Energy). For this intake category, the average % RDA Energy on the heaviest drinking day across men and women stood at 109% (standard deviation (SD) = 36), with the average across all alcohol intake categories at 20% (SD = 29%). Table 3 and Table 4 provide more detailed characteristics for men and women respectively and show that the majority of the sample (88% of men and 82% of women) was not obese (BMI < 30 kg/m^2^). Males had an average BMI of 24.8 kg/m^2^ (SD = 4.8 kg/m^2^). The mean BMI in women was 25.3 kg/m^2^ (SD = 5.8 kg/m^2^). 

In terms of other characteristics, a third of women and a fifth of men had low physical activity levels. Over half the sample (57% men and 50% women) were employed. Around 24% of women and 19% of men reported having a limiting longstanding illness, and 27% of women and 9% of men were parents, with a higher proportion of mothers than fathers in the None category (34% and 10%, respectively).

### 3.2. Relationship between Energy from Alcohol and BMI

Table 5 show the results of the linear regression. With respect to those in the Low category (>0 to 25% RDA Energy), the relationship between alcohol intake and BMI in men showed no differences for intake of alcohol below the Very High (>75% RDA Energy) category. Significant increase in BMI was only observed for the Very High category. For women, however, BMI was significantly associated with % RDA Energy for the High and Very High alcohol intake group (50% and >75% RDA).

**Figure 1:**
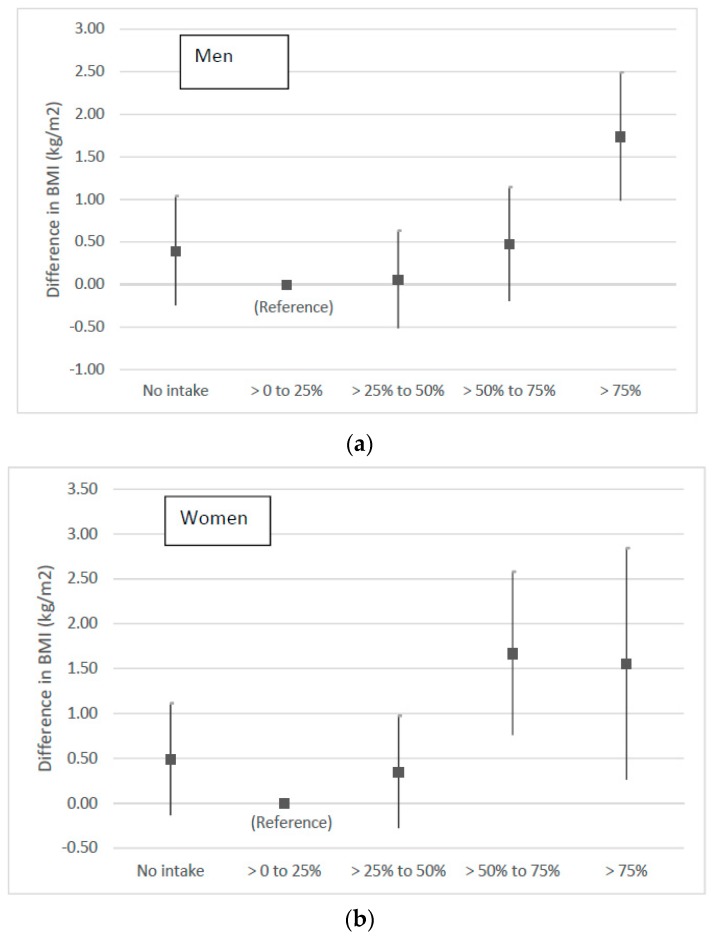
Relationship of BMI to category of intake of energy from alcohol on the heaviest drinking day (%RDA Energy) in men (**a**) and women (**b**).

Figure 1 illustrates these patterns in the results. Further details are provided in the online supplementary material in Table S2.

When analysed by type of alcoholic drink, a relationship with BMI was observed for beer and spirits and alcopops in men, and wine, spirits and alcopops in women, as seen in Table 5. The association between BMI and intake was only significant for the High and Very High intake categories (>50% RDA Energy) for spirits and RTDs. For beer, the difference in BMI was significant only for men reporting High and Very High intakes (>50% of Energy RDA). For wine there was a significant positive association with BMI for the Medium category (>25% to 50%) for men but for women the only significant association was seen once consumption was in the High category (>50% RDA Energy). These associations are shown in the online supplementary material in Tables S3–S5 and graphically represented in in Figures S1–S3.

Vertical bars represent 95% confidence intervals. Estimates of difference in BMI are from a linear regression of BMI on categories of % RDA Energy from total alcoholic beverage intake on the heaviest drinking day. Reference category is Low (>0–25% RDA Energy). All regressions were controlled for age, frequency of intake (number of days the individual had a drink in last 7 days and frequency over the last 12 months), if the individual does not drink alcohol, level of physical activity, employment status, eating more than 5 portions of fruit and vegetables per day, quit smoking in the last year, presence of limiting longstanding illness, ethnicity, being a parent, survey year, and survey country. For an average height in men of 1.75 m, a 0.43 change in the BMI scale is equivalent to 1.32 kg of body weight, and a 1.27 change in BMI translates to 3.9 kg. For a woman of 1.60 m of height, a change in BMI of 0.43 represents a difference of 1.1 kg in weight, and a change of 1.25 BMI units, a weight difference of 3.2 kg.

## 4. Discussion

This study used large national samples from the Health Survey for England and the Scottish Health Survey to explore the relationship between BMI and the contribution to total energy from alcoholic beverage consumption on the heaviest drinking day as a percentage of the recommended daily allowance (RDA) for energy in 18 to 25 year-olds. This measure was used to emphasise the large contribution that alcoholic beverages can make to the gender specific energy RDA and the potential for excessive energy intake on heavy drinking days. Many other studies have used frequency or the average amount of alcohol consumed over a period of a week, but in the study described here the energy consumed in the single heaviest drinking session in the past week was used. This is because a single session potentially has more impact on fat stores as alcohol metabolism inhibits lipid oxidation [9]. Results showed a trend towards a non-linear J-shaped relationship of alcohol intake with BMI, with a significant positive association between alcohol consumption and BMI observed only at Very High levels of intake (>75% RDA Energy) in men and High to Very High intakes (>50% RDA Energy energy) in women. At the same time, in both men and women, the observed relationship between consumption of different types of alcoholic beverages and BMI was similar, The only exception was for beer and women where there was no significant association for any category of % RDA Energy. 

The main results on the relationship between % RDA Energy from alcoholic beverages and BMI found in men are consistent with broader findings in the literature on the association between the amount of alcohol consumed and excess body weight: namely that the amount of alcohol consumed is positively associated with BMI, but that it is higher levels of drinking (excluding alcohol dependence) that significantly influence excess body weight [11,19,20,33,38]. In the case of women, findings of an inverse relationship or a U-shaped relationship between alcohol intake and BMI have been observed more often than in men, possibly driven by higher number of non-drinkers in women generally [15,20,39]. Our results instead showed a positive relationship with alcohol intake that flattened out at High and Very High levels of consumption (>50% RDA Energy), 

It has been suggested that a positive relationship between BMI with high alcohol intake may exist because individuals who consume high levels of alcohol may fail to compensate for the energy intake from alcohol by consuming fewer kcals from food. The evidence for this, however, is stronger for the consumption of spirits and beer than for wine [33,38]. Differences in the relationship between alcohol intake and BMI for different types of drink may be related to differences in dietary and lifestyle patterns associated with consumption of wine, spirits and beer. Wine consumers have been found to eat more fruit and vegetables, and less meat, fried foods, ready-meals, butter, margarine, and eggs, than individuals who mainly drink spirits and beer [40,41,42]. However, our study showed an association with higher BMI for both greater wine and spirits intake, in both men and women, and a positive association with beer intake in men, but not in women. This may be due to the fewer women in the sample drinking a lot of beer, reflecting trends in gender preferences for this alcoholic drink [23,24]. It may also reflect more complex associations between BMI and alcohol intake in women than in men, due to metabolic differences between the sexes [43].

A wealth of epidemiological evidence suggests that non-drinkers have higher BMIs than drinkers [44]. In terms of dietary habits, non-drinkers have been found to consume more energy from carbohydrates than drinkers, which could account for additional differences in body weight [38]. Recent evidence on excess body weight differences between drinkers and abstainers suggests, however, that this effect may be driven by differences in BMI between drinkers and ex-drinkers rather than between drinkers and abstainers as a whole [16]. This is because some ex-drinkers stop consuming alcohol in response to health problems that may affect or be a consequence of high BMI. Results for this study refer to the amount of alcohol consumption controlling for frequency of intake, which implies that this effect may have been attenuated in our results. In fact, in the analyses for the current study no statistically significant differences were found between None and the Low category (the reference level of % RDA Energy). The relatively low proportion of ex-drinkers in the 18 to 25 year group may also have made it difficult to detect any important differences in BMI by level of alcohol intake; only 2% of men 18 to 25 years and 4% of women 18 to 25 years reported being ex-drinkers compared to 6% and 7% for the overall (>18 years) adult sample in the combined HSE and SHeS data.

Both BMI level and alcohol intake may be related to a number of other lifestyle and environmental variables that influence individuals’ weight and alcohol outcomes. An important strength of this study was the ability to take into account a wide range of potential confounders of this relationship, including physical activity, limiting longstanding illness and sociodemographic characteristics of respondents. However, it was not possible to account for food intake associated with alcoholic beverage consumption on the heaviest day of drinking as these data were not collected. It is established that unhealthy food choices are more likely to be made during and directly after a period of excessive alcohol consumption [11]. A further limitation arises from the fact that alcohol intake is likely to be underreported, a phenomena that is has been seen to be more pronounced amongst young males [45]. In addition the data used to calculate energy intakes from alcoholic beverages was obtained from an organisation largely funded by UK alcohol producers, retailers and supermarkets which was chosen as the most suitable available at the time. We have since cross-checked this data with the summarised energy and nutrients supplied to the Living Costs and Food Survey by the Department of Health and Public Health England based on that used for the National Diet and Nutrition Survey. The energy values are compatible with those provided by the organisation [32].

Given the sample size, a limitation of the study was the impossibility of analysing the relationship between alcohol intake and BMI by levels of body overweight. This might have shown stronger associations of BMI with different levels of alcohol intake as some studies suggest that individuals that are already overweight are more prone to weight gain from increased levels of alcohol consumption [10,46]. Moreover, we cannot exclude the possibility that the relationships observed between alcohol consumption and BMI were influenced by confounding from unobserved time-invariant factors related to both drinking and body weight that introduce bias in estimates of associations from cross-sectional analyses. As with other studies in the area, the ability to fully explore the relationship between BMI and the amount of alcohol consumed may have been limited by the low participation of individuals with very high levels of alcohol consumption in health surveys [38]. In addition, the HSE and SHeS response rates for eligible adults range between 59% and 55%, but disaggregated response rates by age groups are not reported, so it is possible that young men and women who are very high alcohol consumers had lower response rates in the survey samples.

The present study provides greater understanding of the likely contribution of alcohol consumption to BMI. Findings show a positive association between BMI and alcohol intake on the heaviest day of drinking but only High and Very High levels of intake (>50% of RDA Energy except for wine in women) are likely to be associated with changes in BMI. Patterns of the relationship of BMI and alcohol intake are similar for consumption of beer, wine, and spirits with slight variations by sex. More longitudinal studies are necessary to confirm these results and elaborate on causal mechanisms at the population level to better explain how alcohol intake impacts BMI. Future work may benefit from including other socio-psychological variables such as personality traits, [47] cultural factors, and policies (e.g., licensing hours) that may predispose to excessive calorie and alcohol consumption.

The findings have several important policy and practice implications. Firstly, for advocacy organisations concerned with alcohol-related harm, or obesity, there may be synergies and opportunities in collaborating. This study further demonstrates that the two problems are interrelated, and it is well-recognised that the policy solutions most likely to be effective-increasing the price of unhealthy commodities, reducing their availability and regulating and reducing marketing exposure–also overlap. Similarly, the political challenges inherent in achieving policy change in these areas may be similar, as action on both is heavily opposed by powerful industry interests. Secondly, for governments developing strategies to reduce obesity and alcohol-related harm, this evidence presents a strong case for such policies to be integrated, or at least strongly cross-referenced. In many countries alcohol policy is combined with policy for illicit drugs, or tobacco, whereas the potential arguments for and synergies from linking alcohol and obesity policy may be (at least) equally strong. 

## Figures and Tables

**Table 1:** Standard volumes and kcal by type of alcoholic drink with percentage of recommended dietary allowance (RDA) for energy (% RDA Energy) of one drink.

Drink Type	Typical Volume (mL) in One Serving	Typical Kcal/mL	Typical Kcal in One Serving	% Contribution of One Serving to RDA Men	% Contribution of One Serving to RDA Women
Normal beer (≤5% alcohol)					
Half pints	284	0.32	91	3.6	4.6
Small cans/bottles	440	0.32	141	5.6	7.1
Large cans/bottles	500	0.32	160	6.4	8.0
Bottles	330	0.32	106	4.2	5.3
Strong beer (≥9% alcohol)					
Half pints	284	0.74	210	8.4	10.5
Small cans/bottles	440	0.74	326	13.0	16.3
Large cans/bottles	500	0.74	370	14.8	18.5
Bottles	330	0.74	244	9.8	12.2
Spirits					
Standard measure	25	2.44	61	2.4	3.1
Sherry					
Standard measure	50	1.26	63	2.5	3.2
Wine					
Small glass	125	0.91	114	4.6	5.7
Medium glass	175	0.91	159	6.4	8.0
Large glass	250	0.91	228	9.1	11.4
Alcopops					
Small cans/bottles	275	0.62	171	6.8	8.6

Adapted from DrinkAware, Alcoholic Drinks and Units. https://www.drinkaware.co.uk/alcohol-facts/alcoholic-drinks-units and NHS UK, Understanding Calories. https://www.nhs.uk/live-well/healthy-weight/understanding-calories/.

**Table 2:** Mean and Median Intake by percentage of recommended daily allowance for energy from alcoholic beverages consumed by individuals on their heaviest drinking day (% RDA energy), percentage of males and females in 18–25 year olds and percentage consuming different types of drink from the combined 2008–2014 Health Survey for England (HSE) and the Scottish Health Survey (SHeS).

	None (0%)	Low (>0–25%)	Medium (>25–50%)	High (>50–75%)	Very High (>75%)	Total
N	3795	2505	1397	635	473	8805
Alcohol (%RDA Energy)						
Mean (SD)	0 (0)	12.5 (6.5)	36.4 (7.1)	60.9 (7.3)	108.5 (36)	19.6 (29.4)
Median (IQR)	0 (0)	12.2 (7.0, 17.8))	35.8 (30.5, 42.6)	60.1 (54.5, 67.9)	97 (84.1, 121.2)	6.4 (0, 29.6)
Sex						
Males% (*n*)	37 (1408)	44 (1096)	48 (667)	58 (368)	61 (299)	44 (3838)
Females % (*n*)	63 (2387)	56 (1409)	52 (730)	42 (267)	39 (174)	56 (4967)
Males						
Alcohol (%RDA Energy)						
Mean (SD)	0 (0)	12.0 (6.5)	36.4 (7.4)	60.9 (7.5)	110 (38)	24.2 (33.6)
Median (IQR)	0 (0)	11.3 (6.4, 17.1)	35.8 (29.1, 43.6)	60.1 (54.0, 67.7)	97 (84.1, 124)	9.8 (0, 37.4)
Drinking status						
Drinker	70 (982)	100 (1096)	100 (667)	100 (368)	100 (299)	89 (3412)
Stopped	5 (69)	0 (0)	0 (0)	0 (0)	0 (0)	2 (69)
Never drinker	25 (357)	0 (0)	0 (0)	0 (0)	0 (0)	9 (357)
Type of drink						
Beer	0 (0)	70 (765)	85 (564)	93 (342)	97 (290)	51 (1961)
Wine	0 (0)	11 (126)	16 (107)	18 (67)	21 (64)	9 (364)
Spirits and RTDs	0 (0)	29 (317)	49 (330)	64 (234)	76 (228)	29 (1109)
Females						
Alcohol (%RDA Energy)						
Mean (SD)	0 (0)	12.9 (6.5)	36.4 (6.7)	60.8 (7.1)	105 (32.0)	16.0 (25.1)
Median (IQR)	0 (0)	12.2 (6.4, 18.2)	34.1 (30.5, 42.2)	60.3 (54.7, 68.2)	95.3 (84.1, 117)	4.5 (0, 24.3)
Drinking status:						
Drinker	72 (1707)	100 (1409)	100 (730)	100 (267)	100 (174)	86 (4287)
Stopped	8 (189)	0 (0)	0 (0)	0 (0)	0 (0)	4 (189)
Never drinker	21 (491)	0 (0)	0 (0)	0 (0)	0 (0)	10 (491)
Type of drink						
Beer	0 (0)	23 (323)	27 (197)	39 (104)	58 (102)	15 (725)
Wine	0 (0)	38 (534)	46 (334)	54 (145)	59 (103)	22 (1116)
Spirits and RTDs	0 (0)	49 (695)	67 (487)	79 (211)	83 (144)	31 (1537)

Unless otherwise stated, rows are percentage values and sample size for the row/column cells are in parenthesis. SD: standard deviation. IQR: inter quartile range. RTDs: ready-to-drink. N is the maximum sample size for each category of %RDA energy. Total numbers may not add up to maximum sample size due to non-response.

**Table 3:** Characteristics of the sample from the combined 2008–2014 HSE and SHS survey, by percentage of recommended daily allowance for energy from alcoholic beverages consumed by individuals on their heaviest drinking day (% RDA Energy) for men.

	None	Low	Medium	High	Very High	
	0%	>0–25%	>25–50%	>50–75%	>75%	Total
Alcohol (%RDA Energy)						
N	1408	1096	667	368	299	3838
BMI						
Mean (SD)	24.7 (5.1)	24.6 (4.8)	24.7 (4.7)	24.9 (4.1)	25.7 (4.7)	24.8 (4.8)
Not obese	85 (1047)	90 (884)	89 (540)	91 (305)	83 (258)	88 (3034)
Obese	15 (179)	10 (99)	11 (69)	9 (29)	17 (52)	12 (428)
Age (years)						
Mean (SD)	21.3 (2.4)	21.8 (2.3)	21.6 (2.2)	21.7 (2.2)	21.1 (2.2)	21.5 (2.3)
Physical activity level						
Low MVPA	24 (249)	21 (177)	16 (82)	17 (50)	17 (46)	21 (604)
Medium MVPA	23 (237)	27 (222)	22 (117)	24 (69)	19 (52)	24 (697)
High MVPA	53 (540)	52 (432)	62 (327)	59 (169)	64 (171)	56 (1639)
Employment status						
Employed	50 (706)	60 (661)	61 (409)	60 (220)	59 (221)	57 (2217)
Unemployed	18 (249)	15 (166)	14 (93)	12 (44)	17 (65)	16 (617)
Other inactive	32 (445)	24 (267)	25 (164)	28 (102)	23 (86)	27 (1064)
FV portions per day % (*n*)						
Less than 5	84 (1070)	83 (839)	83 (506)	87 (298)	87 (312)	84 (3025)
5 or more	16 (202)	17 (176)	17 (101)	13 (44)	13 (45)	16 (568)
Quit smoking						
No/Not applicable	98 (1369)	97 (1056)	96 (642)	96 (354)	98 (298)	97 (3719)
Yes	2 (35)	3 (38)	4 (25)	4 (13)	2 (7)	3 (118)
Longstanding illness:						
No	79 (1112)	82 (899)	83 (554)	83 (306)	80 (314)	81 (3185)
Yes	21 (296)	18 (196)	17 (113)	17 (62)	20 (79)	19 (746)
Ethnicity:						
White and Mixed White	80 (1128)	93 (1023)	97 (646)	99 (364)	97 (362)	90 (3523)
Asian, African, Arab and other	20 (279)	7 (72)	3 (19)	1 (4)	3 (11)	10 (385)
Parent:						
No	90 (1262)	91 (993)	92 (611)	94 (346)	93 (367)	91 (3579)
Yes	10 (146)	9 (103)	8 (56)	6 (22)	7 (27)	9 (354)
Country:						
England	65 (921)	63 (690)	56 (373)	59 (217)	66 (261)	63 (2462)
Scotland	35 (487)	37 (406)	44 (294)	41 (151)	34 (133)	37 (1471)

Unless otherwise stated, rows are percentage values and sample size for the row/column cells are in parenthesis. MVPA: moderate to vigorous physical activity. SD: standard deviation. FV: Fruit and vegetables. Limiting longstanding illness includes mental health and physical conditions affecting vision, hearing, mobility learning and memory, stamina and dexterity (for example, asthma, arthritis, diabetes, cataract, hypertension). N is the maximum sample size for each category of % RDA energy. Total numbers may not add up to maximum sample size due to non-response.

**Table 4:** Characteristics of the sample from the combined 2008–2014 HSE and SHS survey, by percentage of recommended daily allowance for energy from alcoholic beverages consumed by individuals on their heaviest drinking day (% RDA Energy) for women.

	None	Low	Medium	High	Very High	
	0%	>0–25%	>25–50%	>50–75%	>75%	Total
N	2387	1409	730	267	174	4967
BMI:						
Mean (SD)	25.5 (6.1)	25 (5.5)	25 (5.4)	25.9 (5.6)	25.6 (6)	25.3 (5.8)
Not obese	80 (1526)	85 (1059)	86 (555)	79 (190)	81 (151)	82 (3481)
Obese	20 (377)	15 (194)	14 (89)	21 (52)	19 (36)	18 (748)
Age (years):						
Mean (SD)	21.8 (2.3)	21.8 (2.3)	21.6 (2.3)	21.4 (2.3)	20.9 (2.4)	21.7 (2.3)
Physical activity level						
Low MVPA	39 (694)	30 (320)	25 (143)	31 (67)	34 (59)	33 (1283)
Medium MVPA	28 (511)	33 (363)	34 (196)	30 (66)	25 (43)	31 (1179)
High MVPA	33 (595)	37 (401)	41 (234)	39 (85)	41 (70)	36 (1385)
Employment status:						
Employed	45 (1071)	57 (797)	53 (386)	52 (139)	52 (124)	50 (2517)
Unemployed	13 (308)	11 (152)	11 (83)	16 (42)	16 (38)	12 (623)
Other inactive	42 (1002)	32 (456)	36 (260)	32 (86)	33 (78)	37 (1882)
FV portions per day:						
Less than 5	83 (1791)	81 (1040)	84 (570)	85 (205)	87 (195)	83 (3801)
5 or more	17 (376)	19 (247)	16 (108)	15 (37)	13 (29)	17 (797)
Quit smoking:						
No/Not applicable	97 (2304)	97 (1358)	97 (706)	93 (248)	96 (170)	96 (4786)
Yes	3 (79)	3 (49)	3 (22)	7 (19)	4 (7)	4 (176)
Longstanding illness:						
No	75 (1786)	77 (1083)	79 (576)	76 (203)	78 (197)	76 (3845)
Yes	25 (599)	23 (326)	21 (154)	24 (64)	22 (57)	24 (1200)
Ethnicity:						
White and Mixed	83 (1971)	95 (1332)	98 (715)	97 (260)	95 (227)	90 (4505)
Asian, African, Arab and other	17 (415)	5 (76)	2 (15)	3 (7)	5 (13)	10 (526)
Parent:						
No	66 (1569)	79 (1117)	78 (567)	82 (220)	83 (212)	73 (3685)
Yes	34 (818)	21 (292)	22 (163)	18 (47)	17 (43)	27 (1363)
Country:						
England	63 (1506)	61 (861)	55 (405)	60 (160)	65 (167)	61 (3099)
Scotland	37 (881)	39 (548)	45 (325)	40 (107)	35 (88)	39 (1949)

Unless otherwise stated, rows are percentage values and sample size for the row/column cells are in parenthesis. BMI: body mass index. MVPA: moderate to vigorous physical activity. SD: standard deviation. FV: Fruit and vegetables. Limiting longstanding illness includes mental health and physical conditions affecting vision, hearing, mobility learning and memory, stamina and dexterity (for example, asthma, arthritis, diabetes, cataract, hypertension). Total numbers may not add up to maximum sample size due to non-response.

**Table 5:** Relationship between BMI and category of intake of energy from alcohol on the heaviest drinking day (%RDA Energy in categories).

	Males			Females		
	Coef.	*p*-Value	95% CI	Coef.	*p*-Value	95% CI
Total alcohol intake						
No intake	0.40	0.23	−0.25	0.49	0.126	(−0.14, 1.12)
Low > 0 to 25%	(Reference)			(Reference)		
Medium > 25% to 50%	0.06	0.839	(−0.51, 0.63)	0.35	0.278	(−0.28, 0.97)
High > 50% to 75%	0.48	0.164	(−0.19, 1.15)	1.67	<0.001	(0.76, 2.58)
Very High > 75%	1.74	<0.001	(0.98, 2.49)	1.55	0.018	(0.26, 2.85)
Linear trend	0.30	0.004	(0.1, 0.51)	0.33	0.016	(0.06, 0.6)
Frequency (No. of drinks)	−0.04	0.708	(−0.24, 0.16)	-0.15	0.159	(−0.36, 0.06)
Beer intake						
No intake	0.25	0.41	−0.34	-0.40	0.236	(−1.06, 0.26)
Low > 0 to 25%	(Reference)			(Reference)		
Medium > 25% to 50%	0.26	0.371	(−0.31, 0.83)	1.78	0.051	(−0.01, 3.57)
High > 50% to 75%	1.11	0.008	(0.29, 1.94)	0.64	0.637	(−2.02, 3.29)
Very High > 75%	1.58	0.002	(0.59, 2.56)	−0.54	0.592	(−2.51, 1.43)
Linear trend	0.27	0.012	(0.06, 0.47)	0.49	0.016	(0.09, 0.88)
Frequency (No. of drinks)	−0.04	0.678	(−0.24, 0.15)	−0.14	0.178	(−0.33, 0.06)
Wine						
No intake	0.52	0.130	(−0.15, 1.18)	0.47	0.115	(−0.11, 1.05)
Low > 0 to 25%	(Reference)			(Reference)		
Medium > 25% to 50%	1.69	0.022	(0.24, 3.14)	−0.22	0.601	(−1.04, 0.6)
High and Very High > 50%	1.62	0.138	(−0.52, 3.76)	1.80	0.024	(0.24, 3.37)
Linear trend	0.19	0.392	(−0.25, 0.64)	−0.12	0.440	(−0.43, 0.19)
Frequency (No. of drinks)	−0.03	0.767	(−0.22, 0.16)	−0.15	0.129	(−0.34, 0.04)
Spirits						
No intake	0.14	0.572	(−0.35, 0.64)	0.23	0.377	(−0.28, 0.75)
Low > 0 to 25%	(Reference)			(Reference)		
Medium > 25% to 50%	0.47	0.370	(−0.56, 1.5)	0.65	0.150	(−0.24, 1.54)
High and Very High > 50%	1.58	0.037	(0.09, 3.06)	1.58	0.017	(0.29, 2.87)
Linear trend	0.18	0.286	(−0.15, 0.52)	0.28	0.084	(−0.04, 0.59)
Frequency (No. of drinks)	−0.03	0.776	(−0.22, 0.17)	−0.15	0.124	(−0.35, 0.04)

Data from 2008–2014 combined HSE and SHeS data. Estimates of difference in BMI from a linear regression of BMI on categories of % RDA Energy from total alcoholic beverage intake or specific alcoholic beverage. Alcohol intake measured as the total amount consumed on the heaviest drinking day in the last 7 days. All regressions controlled for age, frequency of intake (number of days had a drink in last 7 days and over last 12 months), if the individual does not drink alcohol, level of physical activity, employment status, eating more than 5 portions of fruit and vegetables per day, quit smoking in the last year, presence of limiting longstanding illness, ethnicity, being a parent, survey year and survey country.

## Supplementary Materials

The following are available online at http://www.mdpi.com/2072-6643/10/10/1477/s1, Figure S1: Relationship of BMI to category of intake of energy from beer intake (% RDA Energy) on heaviest drinking day to energy intake by sex, Figure S2: Relationship of BMI to category of intake of energy from wine (%RDA Energy) on heaviest drinking day by sex, Figure S3: Relationship of BMI to category of intake of energy from spirits and RTD (%RDA Energy) on heaviest drinking day by sex, Table S1: Survey year and sample sizes per year used in analyses, Table S2: Regression results for BMI and category of intake of energy from total alcoholic beverages (%RDA Energy) on the heaviest drinking day by sex, Table S3: Regression results for BMI and category of intake of energy from beer intake (%RDA Energy) on heaviest drinking day by sex, Table S4: Regression results for BMI and category of intake of energy from wine intake (% RDA Energy) on heaviest drinking day by sex, Table S5: Regression results for BMI and category of intake of energy from spirits and RTDs intake (% RDA Energy) on heaviest drinking day by sex.

## Abbreviations

BMI	Body Mass Index
RDA	Recommended Daily Allowance
RTDs	Ready-to drink pre-mixed alcoholic beverage such as “alcopops”
HSE	Health Survey for England
SHeS	Scottish Health Survey
FFQ	Food Frequency Questionnaire
IQR	Interquartile range
SD	Standard Deviation
g/day	Grams per day
CI	Confidence Interval
Kcal	Kilocalories
% RDA Energy	Percentage of recommended daily allowance for energy from alcoholic beverages (excluding mixers) on the heaviest drinking day in the last seven days
