# Supplementary material for: Examining Associations between Body Mass Index in 18–25 Year-Olds and Energy Intake from Alcohol: Findings from the Health Survey for England and the Scottish Health Survey
Source: Nutrients. 2018 Oct 10;10(10):1477. doi: 10.3390/nu10101477 (PMC6213174; doi:10.3390/nu10101477)
Supplement: Supplementary file 1 [file nutrients-10-01477-s001.pdf]

Supplementary material

Examining associations between body mass index in 18-25 year-olds and energy intake from alcohol: findings from the Health Survey for England and the Scottish Health Survey

Viviana Albani[1,2], Jennifer Bradley[1,2], Wendy L. Wrieden[1,2,3], Stephanie Scott[3,4], Cassey Muir[1,2], Christine Power[5], Niamh Fitzgerald[6], Martine Stead[6], Eileen Kaner[2,3], Ashley J. Adamson[1,2,3]

viviana.albani@newcastle.ac.uk; jen.bradley@newcastle.ac.uk; wendy.wrieden@newcastle.ac.uk; S.J.Scott@tees.ac.uk; cassey.muir@newcastle.ac.uk; christine.power@ucl.ac.uk; niamh.fitzgerald@stir.ac.uk; martine.stead@stir.ac.uk; eileen.kaner@newcastle.ac.uk; ashley.adamson@newcastle.ac.uk

[1] Human Nutrition Research Centre, Newcastle University, Newcastle upon Tyne, NE2 4HH, UK

[2] Institute of Health and Society, Newcastle University, Newcastle upon Tyne, NE2 4HH,UK

[3]Fuse—the Centre for Translational Research in Public Health, NE2 4HH, UK

[4]School of Social Sciences, Humanities and Law, Teesside University. Middlesbrough, TS13BA, UK

[5]Population, Policy and Practice, UCL Great Ormond Street Institute of Child Health, 30 Guilford Street, London, WC1N 1EH, UK

[6] Institute for Social Marketing, Institute for Social Marketing, UK Centre for Tobacco and Alcohol Studies, Faculty of Health Sciences and Sport, University of Stirling, Stirling, FK9 4LA, UK

Table S1 Survey year and sample sizes per year used in analyses

Year	Health Survey for England	Scottish Health Survey	Total
Including physical activity and fruit and vegetable portions variables			
2008	1,433	491	1,924
2009	.	584	584
2010	.	592	592
2011	.	606	606
2012	.	345	345
2013	604	416	1,020
2014	565	379	944
Total	2,602	3,413	6,015
Including physical activity but excluding fruit and vegetable portions variable			
2008	1,434	492	1,926
2009	.	584	584
2010	.	592	592
2011	.	606	606
2012	769	346	1,115
2013	604	416	1,020
2014	565	379	944
Total	3,372	3,415	6,787
Excluding physical activity and including fruit and vegetable portions variable			
2008	1,439	492	1,931
2009	437	584	1,021
2010	737	593	1,330
2011	758	608	1,366
2012	.	345	345
2013	736	416	1,152
2014	667	379	1,046
Total	4,774	3,417	8,191

Table S2. Regression results for BMI and category of intake of energy from total alcoholic beverages (% RDA Energy) on the heaviest drinking day by sex

	Males				Females			
	Coef.	Std. Err.	p-value	95% CI	Coef.	Std. Err.	p-value	95% CI
None 0%	0.40	0.33	0.23	(-0.25, 1.04)	0.49	0.32	0.126	(-0.14, 1.12)
Low > 0 to 25%	(Reference)				(Reference)			
Medium > 25% to 50%	0.06	0.29	0.839	(-0.51, 0.63)	0.35	0.32	0.278	(-0.28, 0.97)
High > 50% to 75%	0.48	0.34	0.164	(-0.19, 1.15)	1.67	0.46	0.000	(0.76, 2.58)
Very High > 75%	1.74	0.39	0.000	(0.98, 2.49)	1.55	0.66	0.018	(0.26, 2.85)
No. of drinks	-0.04	0.10	0.708	(-0.24, 0.16)	-0.15	0.11	0.159	(-0.36, 0.06)
Alcohol frequency								
>=5 times a week	0.14	0.47	0.757	(-0.77, 1.06)	-0.68	0.82	0.411	(-2.29, 0.94)
1-4 times a week	(Reference)				(Reference)			
1-2 times a month	0.49	0.33	0.139	(-0.16, 1.14)	-0.13	0.28	0.648	(-0.67, 0.41)
<1 every couple of months	-0.07	0.43	0.864	(-0.91, 0.77)	-0.06	0.39	0.872	(-0.83, 0.7)
Stopped drinking	0.20	1.27	0.873	(-2.28, 2.69)	1.70	1.44	0.237	(-1.12, 4.52)
Never-drank	1.18	0.65	0.071	(-0.1, 2.46)	-0.13	0.63	0.831	(-1.36, 1.09)
Age	0.29	0.05	0.000	(0.19, 0.39)	0.23	0.05	0.000	(0.13, 0.34)
Physical activity								
Low MVPA	(Reference)				(Reference)			
Mid MVPA	-0.85	0.38	0.025	(-1.59, -0.11)	-0.38	0.29	0.183	(-0.95, 0.18)
High MVPA	-0.84	0.35	0.018	(-1.53, -0.15)	-1.58	0.30	0.000	(-2.17, -1)
Employment status								
In employment	(Reference)				(Reference)			
Unemployed*	-0.55	0.35	0.116	(-1.25, 0.14)	0.12	0.40	0.769	(-0.66, 0.89)
Other economically inactive	-0.58	0.27	0.028	(-1.1, -0.06)	-0.64	0.24	0.009	(-1.12, -0.16)
> 5 FV portions/day	-0.25	0.24	0.289	(-0.72, 0.21)	-0.16	0.28	0.568	(-0.72, 0.39)
Quit smoking in last 12 months	0.21	0.48	0.669	(-0.74, 1.16)	-0.20	0.60	0.739	(-1.37, 0.97)
Limiting longstanding illness	0.37	0.29	0.198	(-0.19, 0.93)	1.06	0.28	0.000	(0.51, 1.61)

	Males				Females			
	Coef.	Std. Err.	p-value	95% CI	Coef.	Std. Err.	p-value	95% CI
Ethnicity								
White and Mixed	(Reference)				(Reference)			
Indian, Pakistani, Bangladeshi	-1.64	0.58	0.005	(-2.77, -0.51)	-1.49	0.67	0.027	(-2.81, -0.16)
Other Asian	-0.43	0.72	0.546	(-1.84, 0.97)	-4.13	0.47	0.000	(-5.04, -3.21)
African, Arab and Other	-1.13	0.61	0.063	(-2.32, 0.06)	0.44	0.66	0.506	(-0.86, 1.74)
Parent	0.40	0.39	0.307	(-0.36, 1.16)	0.85	0.31	0.005	(0.25, 1.46)
Survey year								
2008	-0.55	0.32	0.087	(-1.19, 0.08)	-0.74	0.36	0.041	(-1.44, -0.03)
2009	-0.49	0.50	0.330	(-1.47, 0.5)	-0.48	0.50	0.339	(-1.45, 0.5)
2010	-0.80	0.50	0.113	(-1.79, 0.19)	-0.26	0.57	0.651	(-1.38, 0.87)
2011	-0.52	0.49	0.283	(-1.48, 0.43)	-0.77	0.54	0.152	(-1.83, 0.29)
2012	-0.36	0.54	0.511	(-1.42, 0.71)	-1.40	0.57	0.015	(-2.52, -0.28)
2013	-0.20	0.37	0.584	(-0.94, 0.53)	0.09	0.41	0.822	(-0.7, 0.89)
2014	(Reference)				(Reference)			
Scotland	0.65	0.29	0.027	(0.08, 1.23)	1.41	0.32	0.000	(0.78, 2.04)
Test of linear trend	0.30	0.105	0.004	(0.1, 0.51)	0.33	0.137	0.016	(0.06, 0.6)

BMI: body mass index. MVPA: moderate to vigorous physical activity. SD: standard deviation. FV: Fruit and vegetables. Limiting longstanding illness includes mental health and physical conditions affecting vision, hearing, mobility learning and memory, stamina and dexterity (for example, asthma, arthritis, diabetes, cataract, hypertension).

Fig S1 Relationship of BMI to category of intake of energy from beer intake on heaviest drinking day (% RDA Energy) to energy intake by sex

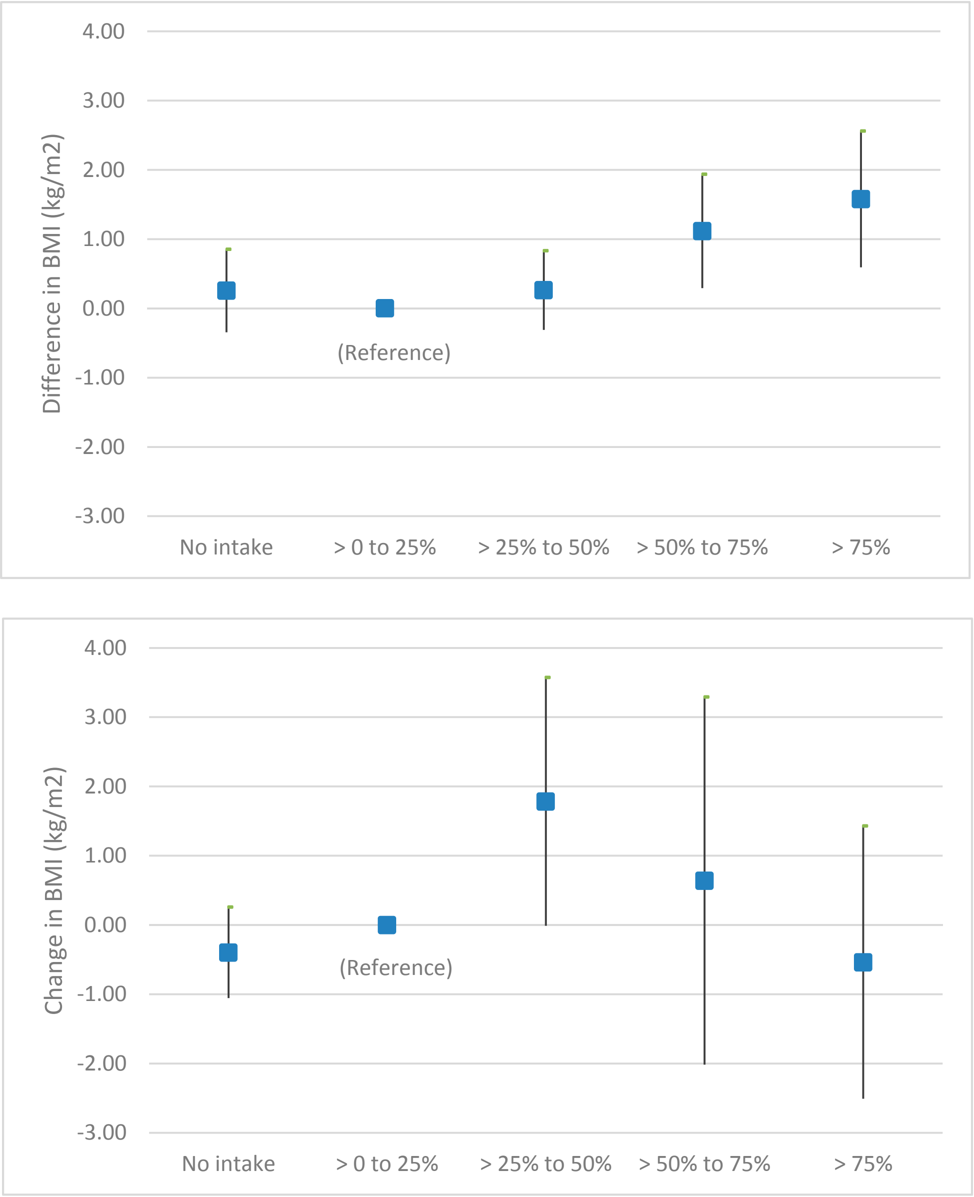

Men (top graph) and women (bottom graph). Vertical bars represent 95% confidence intervals. Estimates of difference in BMI from a linear regression of BMI categories on % RDA Energy from beer intake on the heaviest drinking day. Reference category is > 0 to 25% intake. Alcohol intake measured as the total amount consumed on the heaviest drinking day in the last 7 days. All regressions controlling for age, frequency of intake (number of days had a drink in last 7 days and frequency over the last 12 months), if the individual does not drink alcohol, level of physical activity, employment status, eating more than 5 portions of fruit and vegetables per day, quit smoking in the last year, presence of limiting longstanding illness, ethnicity, being a parent, survey year and survey country.

Table S3. Regression results for BMI and category of intake of energy from beer intake (%RDA Energy) on heaviest drinking day by sex

	**Males**				**Females**			
	Coef.	Std. Err.	p-value	95% CI	Coef.	Std. Err.	p-value	95% CI
None 0%	0.25	0.31	0.41	-0.34	-0.40	0.34	0.236	(-1.06, 0.26)
Low> 0 to 25%	(Reference)				(Reference)			
Medium > 25% to 50%	0.26	0.29	0.371	(-0.31, 0.83)	1.78	0.91	0.051	(-0.01, 3.57)
High > 50% to 75%	1.11	0.42	0.008	(0.29, 1.94)	0.64	1.35	0.637	(-2.02, 3.29)
Very High > 75%	1.58	0.50	0.002	(0.59, 2.56)	-0.54	1.00	0.592	(-2.51, 1.43)
No. of drinks	-0.04	0.10	0.678	(-0.24, 0.15)	-0.14	0.10	0.178	(-0.33, 0.06)
Alcohol frequency								
>=5 times a week	0.18	0.46	0.691	(-0.72, 1.09)	-0.71	0.79	0.372	(-2.26, 0.85)
1-4 times a week	(Reference)				(Reference)			
1-2 times a month	0.51	0.33	0.121	(-0.13, 1.15)	-0.15	0.28	0.593	(-0.69, 0.4)
<1 every couple of months	-0.02	0.43	0.970	(-0.86, 0.83)	0.01	0.39	0.989	(-0.76, 0.78)
Stopped drinking	0.33	1.26	0.793	(-2.14, 2.8)	1.88	1.51	0.214	(-1.08, 4.84)
Never-drank	1.30	0.65	0.046	(0.02, 2.58)	0.02	0.63	0.973	(-1.21, 1.26)
Other drinks	-0.06	0.20	0.747	(-0.45, 0.32)	-0.18	0.20	0.370	(-0.57, 0.21)
Age	0.28	0.05	0.000	(0.18, 0.38)	0.22	0.05	0.000	(0.11, 0.33)
Physical activity								
Low MVPA	(Reference)				(Reference)			
Mid MVPA	-0.83	0.38	0.028	(-1.57, -0.09)	-0.40	0.30	0.190	(-0.99, 0.2)
High MVPA	-0.78	0.35	0.026	(-1.47, -0.09)	-1.56	0.31	0.000	(-2.16, -0.96)
Employment status								
In employment	(Reference)				(Reference)			
Unemployed*	-0.52	0.35	0.141	(-1.21, 0.17)	0.15	0.39	0.697	(-0.61, 0.92)
Other economically inactive	-0.59	0.27	0.028	(-1.11, -0.06)	-0.67	0.25	0.009	(-1.16, -0.17)
> 5 FV portions/day	-0.24	0.24	0.301	(-0.7, 0.22)	-0.19	0.29	0.513	(-0.76, 0.38)
Quit smoking in last 12 months	0.17	0.49	0.725	(-0.79, 1.14)	-0.14	0.58	0.806	(-1.28, 1)
Limiting longstanding illness	0.38	0.29	0.186	(-0.18, 0.95)	1.12	0.28	0.000	(0.58, 1.66)

	Males				Females			
	Coef.	Std. Err.	p-value	95% CI	Coef.	Std. Err.	p-value	95% CI
Ethnicity								
White and Mixed	(Reference)				(Reference)			
Indian, Pakistani, Bangladeshi	-1.64	0.58	0.005	(-2.78, -0.51)	-1.48	0.66	0.025	(-2.77, -0.19)
Other Asian	-0.45	0.72	0.527	(-1.85, 0.95)	-4.15	0.47	0.000	(-5.08, -3.23)
African, Arab and Other	-1.13	0.61	0.062	(-2.32, 0.06)	0.39	0.66	0.556	(-0.91, 1.69)
Parent	0.37	0.39	0.348	(-0.4, 1.13)	0.85	0.31	0.006	(0.25, 1.46)
Survey year								
2008	-0.56	0.32	0.082	(-1.2, 0.07)	-0.63	0.36	0.079	(-1.33, 0.07)
2009	-0.48	0.50	0.342	(-1.46, 0.5)	-0.48	0.53	0.366	(-1.51, 0.56)
2010	-0.81	0.51	0.114	(-1.81, 0.19)	-0.29	0.55	0.600	(-1.37, 0.79)
2011	-0.55	0.49	0.258	(-1.51, 0.4)	-0.78	0.53	0.142	(-1.81, 0.26)
2012	-0.37	0.55	0.492	(-1.45, 0.7)	-1.45	0.60	0.015	(-2.62, -0.28)
2013	-0.25	0.37	0.506	(-0.98, 0.48)	0.08	0.39	0.842	(-0.69, 0.85)
2014	(Reference)				(Reference)			
Scotland	0.66	0.29	0.024	(0.09, 1.23)	1.49	0.32	0.000	(0.86, 2.12)
Test of linear trend	0.27	0.105	0.012	(0.06, 0.47)	0.49	0.202	0.016	(0.09, 0.88)

BMI: body mass index. MVPA: moderate to vigorous physical activity. SD: standard deviation. FV: Fruit and vegetables. Limiting longstanding illness includes mental health and physical conditions affecting vision, hearing, mobility learning and memory, stamina and dexterity (for example, asthma, arthritis, diabetes, cataract, hypertension).

Fig S2 Relationship of BMI to category of intake of energy (%RDA Energy) from wine on heaviest drinking day by sex

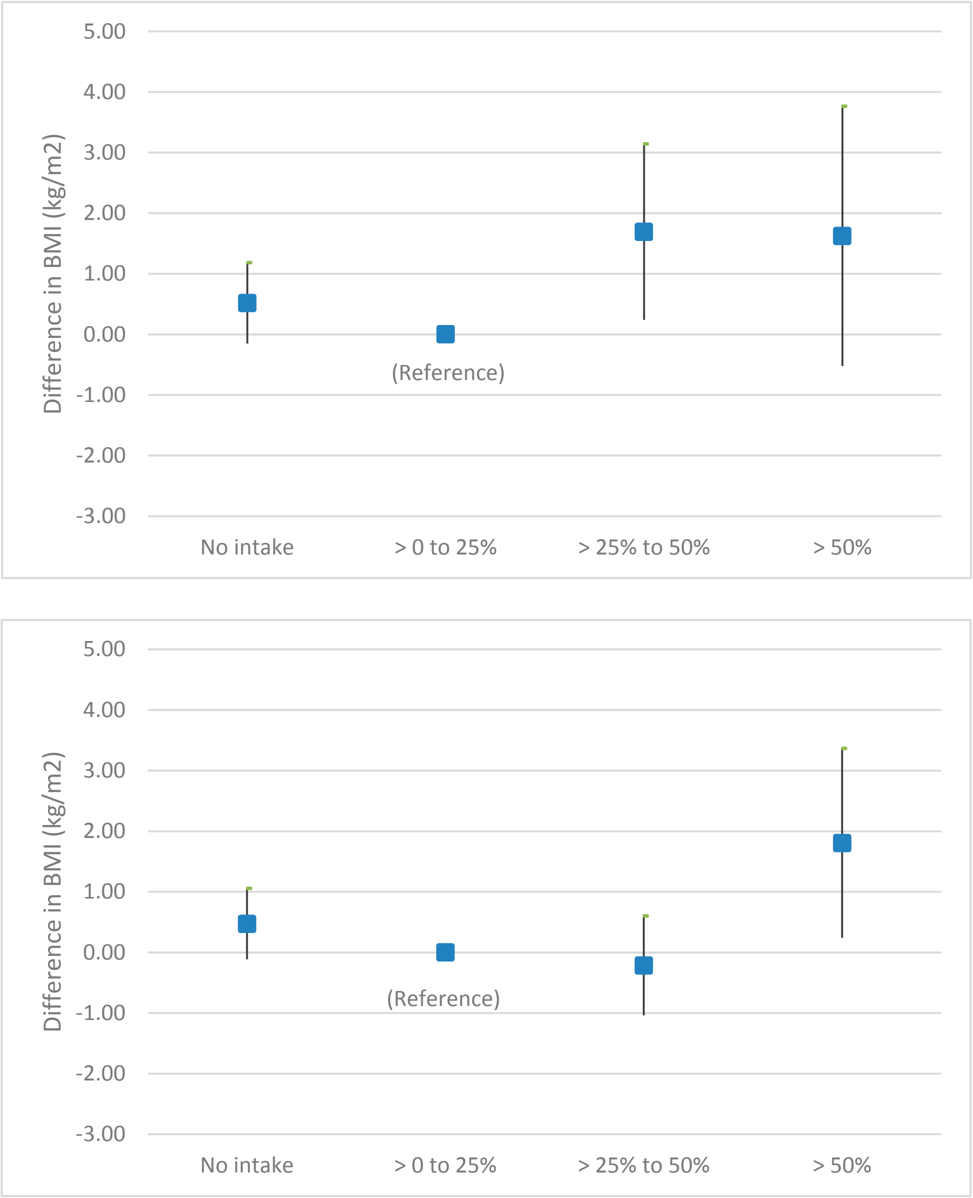

Men (top graph) and women (bottom graph). Vertical bars represent 95% confidence intervals. Estimates of difference in BMI from a linear regression of BMI on categories of % RDA Energy from wine on the heaviest drinking day. Reference category is > 0 to 25% intake. All regressions controlling for age, frequency of intake (number of days had a drink in last 7 days and frequency over the last 12 months), if the individual does not drink alcohol, level of physical activity, employment status, eating more than 5 portions of fruit and vegetables per day, quit smoking in the last year, presence of limiting longstanding illness, ethnicity, being a parent, survey year and survey country.

Table S4. Regression results for BMI and category of intake of energy from wine intake (% RDA Energy) on heaviest drinking day by sex

	Males				Females			
	Coef.	Std. Err.	p-value	95% CI	Coef.	Std. Err.	p-value	95% CI
Alcohol intake (%RDA Energy)								
None 0%	0.52	0.34	0.130	(-0.15, 1.18)	0.47	0.30	0.115	(-0.11, 1.05)
Low > 0 to 25%	(Reference)				(Reference)			
Medium > 25% to 50%	1.69	0.74	0.022	(0.24, 3.14)	-0.22	0.42	0.601	(-1.04, 0.6)
High/Very High > 50%	1.62	1.09	0.138	(-0.52, 3.76)	1.80	0.80	0.024	(0.24, 3.37)
No. of drinks	-0.03	0.10	0.767	(-0.22, 0.16)	-0.15	0.10	0.129	(-0.34, 0.04)
Alcohol frequency								
>=5 times a week	0.21	0.46	0.655	(-0.7, 1.12)	-0.54	0.82	0.513	(-2.14, 1.07)
1-4 times a week	(Reference)				(Reference)			
1-2 times a month	0.47	0.33	0.159	(-0.18, 1.12)	-0.17	0.28	0.548	(-0.71, 0.38)
<1 every couple of months	-0.05	0.43	0.902	(-0.9, 0.79)	-0.01	0.39	0.987	(-0.77, 0.76)
Stopped drinking	0.33	1.26	0.795	(-2.14, 2.8)	1.85	1.51	0.222	(-1.12, 4.81)
Never-drank	1.35	0.65	0.038	(0.08, 2.62)	0.00	0.63	0.994	(-1.23, 1.24)
Other drinks	0.03	0.18	0.891	(-0.33, 0.38)	0.32	0.20	0.111	(-0.07, 0.72)
Age	0.28	0.05	0.000	(0.18, 0.38)	0.24	0.05	0.000	(0.13, 0.35)
Physical activity								
Low MVPA	(Reference)				(Reference)			
Mid MVPA	-0.82	0.38	0.032	(-1.56, -0.07)	-0.39	0.30	0.196	(-0.98, 0.2)
High MVPA	-0.76	0.35	0.031	(-1.45, -0.07)	-1.58	0.31	0.000	(-2.18, -0.98)
Employment status								
In employment	(Reference)				(Reference)			
Unemployed*	-0.54	0.36	0.129	(-1.24, 0.16)	0.21	0.39	0.592	(-0.55, 0.97)
Other economically inactive	-0.64	0.27	0.017	(-1.16, -0.11)	-0.64	0.25	0.012	(-1.14, -0.14)
> 5 FV portions/day	-0.28	0.24	0.232	(-0.75, 0.18)	-0.17	0.29	0.554	(-0.74, 0.39)
Quit smoking in last 12 months	0.14	0.49	0.776	(-0.82, 1.1)	-0.21	0.58	0.709	(-1.34, 0.91)

	Males				Females			
	Coef.	Std. Err.	p-value	95% CI	Coef.	Std. Err.	p-value	95% CI
Limiting longstanding illness	0.41	0.29	0.152	(-0.15, 0.98)	1.10	0.28	0.000	(0.56, 1.64)
Ethnicity								
White and Mixed	(Reference)				(Reference)			
Indian, Pakistani, Bangladeshi	-1.72	0.58	0.003	(-2.86, -0.58)	-1.49	0.66	0.023	(-2.78, -0.2)
Other Asian	-0.57	0.70	0.420	(-1.95, 0.81)	-4.16	0.47	0.000	(-5.08, -3.25)
African, Arab and Other	-1.14	0.60	0.058	(-2.32, 0.04)	0.38	0.66	0.566	(-0.92, 1.68)
Parent	0.41	0.39	0.294	(-0.35, 1.17)	0.86	0.31	0.006	(0.25, 1.47)
Survey year								
2008	-0.45	0.32	0.166	(-1.08, 0.18)	-0.67	0.36	0.065	(-1.37, 0.04)
2009	-0.37	0.51	0.466	(-1.36, 0.62)	-0.55	0.53	0.297	(-1.59, 0.49)
2010	-0.62	0.51	0.223	(-1.63, 0.38)	-0.33	0.55	0.547	(-1.4, 0.74)
2011	-0.45	0.49	0.361	(-1.41, 0.51)	-0.85	0.53	0.109	(-1.88, 0.19)
2012	-0.24	0.55	0.658	(-1.31, 0.83)	-1.41	0.60	0.018	(-2.58, -0.24)
2013	-0.19	0.37	0.609	(-0.92, 0.54)	0.08	0.39	0.843	(-0.69, 0.84)
2014	(Reference)				(Reference)			
Scotland	0.58	0.29	0.049	(0, 1.16)	1.43	0.33	0.000	(0.79, 2.07)
Test of linear trend	0.19	0.226	0.392	(-0.25, 0.64)	-0.12	0.156	0.440	(-0.43, 0.19)

BMI: body mass index. MVPA: moderate to vigorous physical activity. SD: standard deviation. FV: Fruit and vegetables. Limiting longstanding illness includes mental health and physical conditions affecting vision, hearing, mobility learning and memory, stamina and dexterity (for example, asthma, arthritis, diabetes, cataract, hypertension).

Fig S3. Relationship of BMI to category of intake of energy from spirits and RTDs on heaviest drinking day (% RDA Energy) by sex.

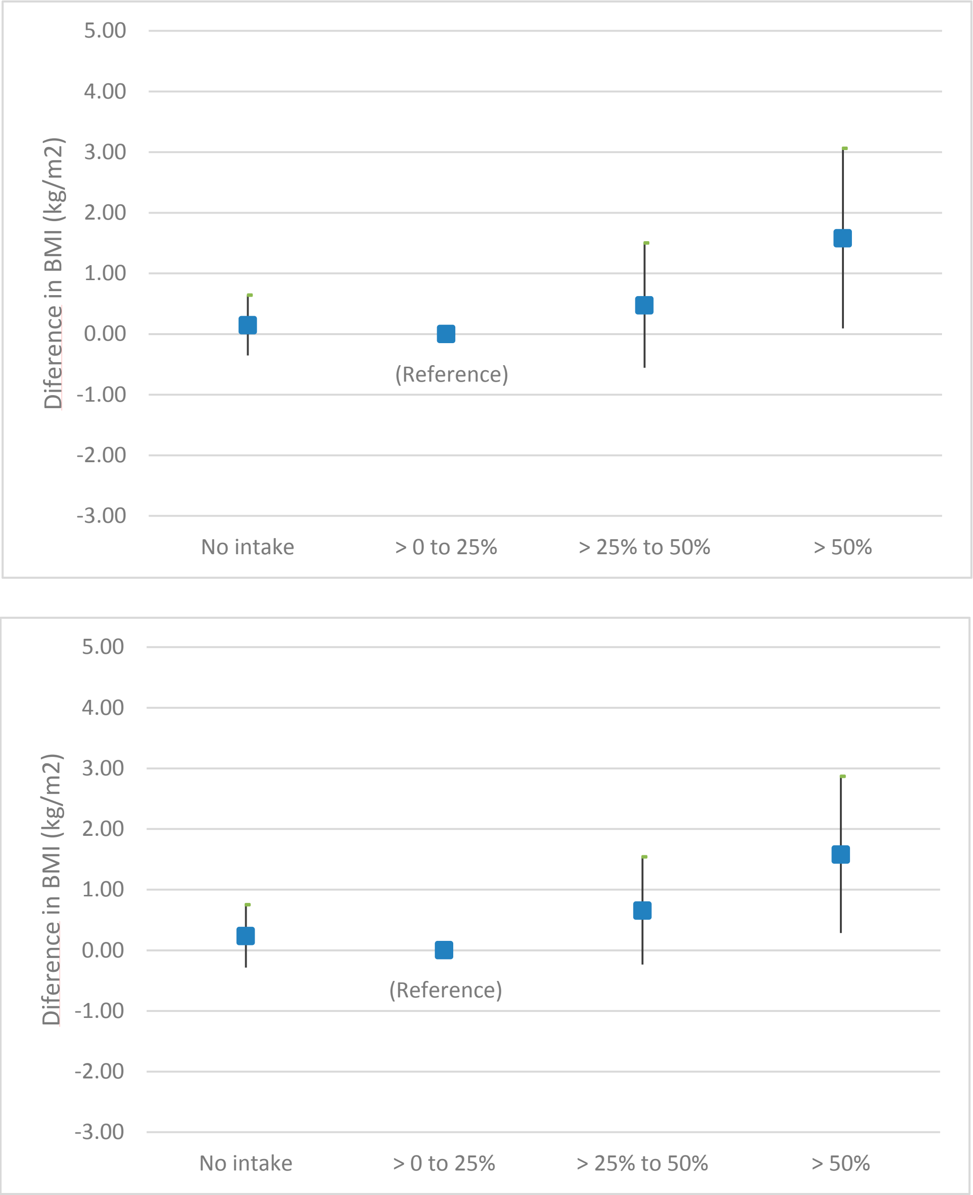

Men (top graph) and women (bottom graph). Vertical bars represent 95% confidence intervals. Estimates of difference in BMI from a linear regression of BMI on categories of % RDA Energy from spirits and RTDs intake on the heaviest drinking day. Reference category is > 0 to 25% intake. All regressions controlling for age, frequency of intake (number of days had a drink in last 7 days and frequency over the last 12 months), if the individual does not drink alcohol, level of physical activity, employment status, eating more than 5 portions of fruit and vegetables per day, quit smoking in the last year, presence of limiting longstanding illness, ethnicity, being a parent, survey year and survey country.

Table S5. Regression results for BMI and category of intake of energy from spirits and RTDs intake (% energy RDA) on heaviest drinking day by sex

	Males				Females			
	Coef.	Std. Err.	p-value	95% CI	Coef.	Std. Err.	p-value	95% CI
Alcohol intake (% RDA Energy)	0.14	0.25	0.572	(-0.35, 0.64)				
None 0%	(Reference)				0.23	0.26	0.377	(-0.28, 0.75)
Low > 0 to 25%	0.47	0.53	0.370	(-0.56, 1.5)	(Reference)			
Medium > 25% to 50%	1.58	0.76	0.037	(0.09, 3.06)	0.65	0.45	0.150	(-0.24, 1.54)
High/Very High > 50%	0.14	0.25	0.572	(-0.35, 0.64)	1.58	0.66	0.017	(0.29, 2.87)
No. of drinks	-0.03	0.10	0.776	(-0.22, 0.17)	-0.15	0.10	0.124	(-0.35, 0.04)
Alcohol frequency								
>=5 times a week	0.22	0.46	0.637	(-0.69, 1.12)	-0.50	0.80	0.531	(-2.06, 1.06)
1-4 times a week	(Reference)				(Reference)			
1-2 times a month	0.45	0.33	0.175	(-0.2, 1.09)	-0.11	0.28	0.699	(-0.65, 0.44)
<1 every couple of months	-0.05	0.43	0.910	(-0.89, 0.79)	0.05	0.39	0.903	(-0.72, 0.81)
Stopped drinking	0.32	1.26	0.800	(-2.15, 2.79)	1.94	1.51	0.199	(-1.02, 4.9)
Never-drank	1.32	0.65	0.042	(0.05, 2.6)	0.07	0.63	0.908	(-1.17, 1.31)
Other drinks	0.02	0.23	0.932	(-0.44, 0.48)	0.12	0.22	0.576	(-0.31, 0.55)
Age	0.29	0.05	0.000	(0.19, 0.39)	0.23	0.06	0.000	(0.12, 0.34)
Physical activity								
Low MVPA	(Reference)				(Reference)			
Mid MVPA	-0.83	0.38	0.028	(-1.57, -0.09)	-0.40	0.30	0.186	(-1, 0.19)
High MVPA	-0.79	0.35	0.025	(-1.49, -0.1)	-1.57	0.31	0.000	(-2.17, -0.97)
Employment status								
In employment	(Reference)				(Reference)			
Unemployed*	-0.52	0.36	0.143	(-1.22, 0.18)	0.15	0.39	0.699	(-0.62, 0.92)
Other economically inactive	-0.61	0.26	0.021	(-1.13, -0.09)	-0.63	0.26	0.014	(-1.13, -0.13)
> 5 FV portions/day	-0.30	0.24	0.203	(-0.77, 0.16)	-0.18	0.29	0.518	(-0.75, 0.38)
Quit smoking in last 12 months	0.20	0.50	0.685	(-0.77, 1.17)	-0.10	0.57	0.858	(-1.21, 1.01)

	Males				Females			
	Coef.	Std. Err.	p-value	95% CI	Coef.	Std. Err.	p-value	95% CI
Limiting longstanding illness	0.42	0.29	0.150	(-0.15, 0.99)	1.07	0.28	0.000	(0.53, 1.61)
Ethnicity								
White and Mixed	(Reference)				(Reference)			
Indian, Pakistani, Bangladeshi	-1.67	0.58	0.004	(-2.81, -0.53)	-1.51	0.66	0.023	(-2.8, -0.21)
Other Asian	-0.48	0.72	0.500	(-1.89, 0.92)	-4.16	0.47	0.000	(-5.08, -3.23)
African, Arab and Other	-1.13	0.61	0.062	(-2.32, 0.06)	0.42	0.67	0.528	(-0.89, 1.73)
Parent	0.42	0.39	0.283	(-0.35, 1.19)	0.82	0.31	0.009	(0.21, 1.44)
Survey year								
2008	-0.53	0.32	0.104	(-1.16, 0.11)	-0.71	0.36	0.049	(-1.42, 0)
2009	-0.47	0.50	0.353	(-1.46, 0.52)	-0.50	0.53	0.345	(-1.54, 0.54)
2010	-0.77	0.50	0.119	(-1.75, 0.2)	-0.25	0.55	0.645	(-1.32, 0.82)
2011	-0.54	0.49	0.270	(-1.5, 0.42)	-0.77	0.53	0.149	(-1.81, 0.27)
2012	-0.32	0.55	0.558	(-1.39, 0.75)	-1.37	0.60	0.021	(-2.54, -0.2)
2013	-0.24	0.37	0.512	(-0.97, 0.48)	0.09	0.39	0.825	(-0.68, 0.85)
2014	(Reference)				(Reference)			
Scotland	0.63	0.29	0.030	(0.06, 1.21)	1.42	0.33	0.000	(0.78, 2.06)
Test of linear trend	0.18	0.170	0.286	(-0.15, 0.52)	0.28	0.161	0.084	(-0.04, 0.59)
